# An Enriched Environment Alters DNA Repair and Inflammatory Responses After Radiation Exposure

**DOI:** 10.3389/fimmu.2021.760322

**Published:** 2021-10-22

**Authors:** Sae Sakama, Keisuke Kurusu, Mayu Morita, Takashi Oizumi, Shinya Masugata, Shohei Oka, Shinya Yokomizo, Mayumi Nishimura, Takamitsu Morioka, Shizuko Kakinuma, Yoshiya Shimada, Asako J. Nakamura

**Affiliations:** ^1^ Department of Biological Science, College of Sciences, Ibaraki University, Mito, Japan; ^2^ Department of Radiation Effects Research, National Institute of Radiological Sciences (NIRS), National Institutes for Quantum and Radiological Science and Technology (QST), Chiba, Japan; ^3^ Executive Director, National Institutes for Quantum and Radiological Science and Technology (QST), Chiba, Japan

**Keywords:** enriched environment (EE), DNA damage, histone H2AX, inflammation, macrophages, radiation-induced carcinogenesis

## Abstract

After the Fukushima Daiichi Nuclear Power Plant accident, there is growing concern about radiation-induced carcinogenesis. In addition, living in a long-term shelter or temporary housing due to disasters might cause unpleasant stress, which adversely affects physical and mental health. It’s been experimentally demonstrated that “eustress”, which is rich and comfortable, has beneficial effects for health using mouse models. In a previous study, mice raised in the enriched environment (EE) has shown effects such as suppression of tumor growth and enhancement of drug sensitivity during cancer treatment. However, it’s not yet been evaluated whether EE affects radiation-induced carcinogenesis. Therefore, to evaluate whether EE suppresses a radiation-induced carcinogenesis after radiation exposure, in this study, we assessed the serum leptin levels, radiation-induced DNA damage response and inflammatory response using the mouse model. In brief, serum and tissues were collected and analyzed over time in irradiated mice after manipulating the raising environment during the juvenile or adult stage. To assess the radiation-induced DNA damage response, we performed immunostaining for phosphorylated H2AX which is a marker of DNA double-strand break. Focusing on the polarization of macrophages in the inflammatory reaction that has an important role in carcinogenesis, we performed analysis using tissue immunofluorescence staining and RT-qPCR. Our data confirmed that EE breeding before radiation exposure improved the responsiveness to radiation-induced DNA damage and basal immunity, further suppressing the chronic inflammatory response, and that might lead to a reduction of the risk of radiation-induced carcinogenesis.

## Introduction

Social concern about the effects of radiation exposure on the human body has grown in recent years owing to the accident at the Fukushima Daiichi Nuclear Power Plant and to the presence of human beings in outer space. Radiation exposure can cause psychological and physical effects. A questionnaire survey of atomic bomb survivors approximately 50 years after exposure reported that their physical and mental health deteriorated in proportion to the distance from the epicenter of the atomic bomb explosion (1). In addition, neighboring residents were often forced to live in evacuation shelters or temporary housing for long periods of time, leading to major changes in their living environment. Sleep, exercise, and social ties have become insufficient owing to this accident, and deteriorating health resulting from these increased psychological and physical stresses have occurred. In the case of space flight, stress from a closed environment owing to long-term stays in the International Space Station (ISS) during a mission is also seen as a problem (2). Stress and mental state are known to affect the immune system, and there are many reports suggesting that increased inflammatory molecules and decreased T cell activity promote the onset and progression of cancer (3–6). Previous studies have proposed an experimental system called the enriched environment (EE) to investigate how the physical and social environment is associated with disease risk and progression (7–10). EE has a larger cage volume than the standard environment (SE) used in general animal experiments, and the exercise and social stimulation made available to the domestic animals create a comfortable environment where “eustress” is given. EE has been reported to have health-promoting effects, such as increased drug sensitivity during cancer treatment and reduced anxiety-like behavior (11–14). EE is also known to suppress tumor growth by increasing the production of brain-derived neurotrophic factor (BDNF) in the hypothalamus, which reduces leptin production *via* sympathetic β-adrenergic receptors (15). Although several reports have indicated that improved living environments inhibit tumor growth, whether EE affects radiation-induced carcinogenesis is unclear.

The carcinogenic process owing to radiation exposure progresses with the complex activation of various cell and tissue responses, and the DNA damage response is one of the initial events. Among the types of radiation-induced DNA damage, double-strand breaks (DSBs) are severe lesions that may induce cell death, cellular senescence, and tumorigenesis (16). However, cells have several DNA repair mechanisms that maintain genomic stability. H2AX, a variant of histone H2A, is phosphorylated around the damage site when DSB occurs. Phosphorylated H2AX (γ-H2AX) can be detected as a focus at the damage site and promotes the recruitment of DNA damage repair proteins (17, 18). In addition to the DNA damage response, the inflammatory response is a critical response induced after radiation exposure, and this is an important biological response in the carcinogenic process. Inflammation is divided into two types: acute inflammation, which subsides in a short period of time, and chronic inflammation, which persists for a long period of time. This type of inflammation is closely related to macrophage polarization and function (19–22). Pro-inflammatory M1 macrophages produce inflammatory cytokines and exert antibacterial effects. Anti-inflammatory M2 macrophages are accompanied by changes in the tissue microenvironment because they not only have anti-inflammatory effects but also angiogenesis and tissue repair effects (23–26). Therefore, effective control of the macrophage M1/M2 polarization results in a quick resolution of the inflammatory response and restoration of the normal tissue structure. However, chronic inflammation induced by activated macrophages is thought to increase the risk of carcinogenesis by causing excessive tissue damage and changes in the tissue environment that favor the growth of cancer cells (20, 27–30).

Therefore, to clarify whether the inhibitory effect of EE on tumor growth persists even after radiation exposure, in this study we focused on DNA damage repair and the inflammatory response, which are heavily involved in the radiation-induced carcinogenesis process, and investigated the effects of EE over time. We also analyzed the effects of EE breeding in juveniles and adults. Our results show that EE might lead to a reduction of the risk of radiation-induced carcinogenesis.

## Materials and Methods

### Mice

For all the experiments, we used 3-week-old B6C3F1 male mice purchased from Charles River Japan. Animal care and experimental schedules were approved by the National Institute of Radiological Sciences (NIRS), National Institutes for Quantum and Radiological Science and Technology (QST) of Japan and were in strict accordance with the guidelines of the Institute.

### Enriched Environment

We prepared standard environment (SE) and enriched environment (EE) as previously described (31). Briefly, the EE cage is larger in volume than the SE cage (W×L×H – 300 mm×170 mm×110 mm) and has enriched environment maintenance supplies: EE cage (W×L×H – 426 mm×542 mm×200 mm). Five mice per cage were housed in EE or SE cages.

### Housing Conditions

Both mice groups, SE and EE were housed under a room temperature (23 ± 1°C) and humidity (45 ± 5%) with a 12 h-light/12 h-dark cycles, and had free access to water and food. The purchased mice were divided into 4 groups for the experiment. The 1st group is a group that 3-week-old mice housed in SE cage for 8 weeks (hereinafter called Juvenile SE). The 2nd group is a group that 3-week-old mice housed in EE cage for 8 weeks (hereinafter called Juvenile EE). The 3rd group is a group that 3-week-old mice housed in SE cage for 16 weeks (hereinafter called Adult SE). The 4th group is a group that 3-week-old mice housed in SE cage for 8 weeks and then 11-week-old mice housed in EE cage for 8 weeks (hereinafter called Adult SE).

### Irradiation and Sampling

The juvenile SE and juvenile EE groups were irradiated with 0 or 2 Gy of X-rays at 11 weeks of age, and the adult SE and adult EE groups were irradiated with 0 or 2 Gy of X-rays at 19 weeks of age and were euthanized by exsanguination under deep anesthesia with 4% isoflurane over time (0, 1, 3, 6, 24, 144 hours after irradiation). Serum and organs were harvested from three mice at each time point. Serum was cryopreserved, organs were frozen in OCT compound with liquid nitrogen. The work up to this point was done at QST (Chiba, Japan) and these sample were stored in a J-SHARE archive (32). After that, a part of the archive sample was provided to Ibaraki University. The delivered frozen sample was stored at -80°C.

### Immunohistochemistry (Frozen Section)

Immunohistochemical (IHC) staining was performed using frozen tissue sections. Frozen organs were cut into 4 μm sections and the sections were placed on glass slides overnight at room temperature. Sections were hydrophilized with PBS at room temperature for 15 min, fixed with 4% paraformaldehyde at room temperature for 20 min, and then permeabilized with 70% ethanol overnight at 4°C. The sections were blocked with 5% bovine serum albumin, 0.5% Tween20, and 0.1% TritonX-100 in PBS for 1 h at room temperature after hydrophilized with PBS for 15 min at room temperature. The tissue sections were then immunostained with the following antibodies: anti-γ-H2AX (1:2000, NB100-384, Novus Biologicals) and anti-F4/80 (1:500, ab6640, abcam). As the secondary antibody, antibodies conjugated with Alexa488 (1:700, A11006, Thermo Fisher Scientific) was used. Finally, a mounting medium containing propidium iodide (Vector Laboratories) or DAPI (Vector Laboratories) was used. Fluorescence was detected using a fluorescence microscope (BX53, Olympus, Tokyo, Japan) and analyzed after capturing digital images using Cellsens software.

### Evaluation of γ-H2AX-Positive Cells

Analysis was performed based on the acquired digital image. Cells with 4 or more γ-H2AX foci per cell were defined as γ-H2AX-positive cells and at least 500 cells were randomly measured before calculating the ratio. The decrease in the positive cell rate relative to the γ-H2AX-positive cell rate detected 1 h after irradiation was calculated as the repair efficiency of DNA damage.

### Evaluation of F4/80 Positive Intensity

Analysis was performed based on the acquired digital image. For lung tissue, the F4/80 intensity grade per field was visually evaluated in four levels (negative and positive 1, 2, or 3). For liver tissue, ImageJ was used to measure the percentage of F4/80-positive area per visual field in each sample.

### RT-qPCR

Total RNA was isolated from frozen OCT compound sections using the RNeasy Plus Micro Kit (Qiagen, Hilden, Germany) according to the manufacturer’s protocol. A reverse transcriptase reaction was performed using SuperScript IV VILO Master Mix with ezDNase enzyme (Invitrogen, Carlsbad, CA) according to the manufacturer’s protocol. Using the synthesized cDNA as a template, *TNF* (Mm00443258 m1) and *Arg1* (Mm00475988 m1) mRNA expression levels were quantified using TaqMan^®^ Gene Expression Assays (Applied Biosystems, Foster City, CA) and Step One real-time PCR system (Applied Biosystems, Foster City, CA). Cycling conditions were set according to manufacturer’s instructions. Relative expression levels were determined by applying comparative C_T_ methods using *GAPDH* (Mm99999915 g1) as an endogenous control.

### ELISA

Leptin levels in mouse serum were determined using the Leptin Mouse ELISA Kit (abcam, Cambridge, UK) according to the manufacturer’s protocol. Mouse serum was used in a 10-fold dilution.

### Statistical Analysis

Data from the ELISA and the IHC staining were expressed as the mean ± standard error of the mean (SEM). Data from the RT-qPCR were expressed as the mean ± standard deviation (SD). The significance of the data was evaluated by means of the *F*-test and *t*-test. Student’s t-test (two-tailed) or Welch’s t-test (two-tailed) was performed according to the results of the *F*-test *via* the Microsoft Excel. A *P* value of < 0.05 was considered statistically significant.

## Results

### EE Reduces Leptin Concentrations Even After Radiation Exposure

**Figure 1:**
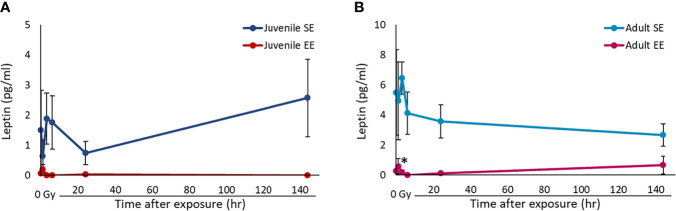
Effect of EE on serum leptin concentration. **(A, B)** Changes in leptin concentration over time after X-ray non-irradiation and 2 Gy irradiation of juvenile SE/EE **(A)** or adult SE/EE **(B)** mice. The data show mean ± SEM; n = 3 mice per group. The statistical approach is Student’s t-test (two-tailed) (Juvenile group; 1 h after irradiation. Adult group; 1, 144 h after irradiation) or Welch’s t-test (two-tailed) (Juvenile group; before irradiation and 3-144 h after irradiation. Adult group; before irradiation and 3-24 h after irradiation.) was performed according to the results of the F-test *via* the Microsoft Excel. *P < 0.05 compared to the SE group.

To examine whether the influence of EE depends on the timing of EE breeding, two groups were set: a juvenile group in which the breeding environment was manipulated from 3 weeks of age and an adult group in which the breeding environment was manipulated from 11 weeks of age. By setting two groups in this way, we examined the effects of EE breeding in juveniles and adults. Previous studies have shown that serum leptin concentrations decrease in mice raised in an EE (15). However, it is unclear whether this effect depends on the timing of EE breeding and whether it persists after radiation exposure. We used serum collected from mice in all experimental groups and ELISA to investigate the effect of EE on leptin protein levels. Importantly, it was clarified that the serum leptin concentrations decrease without irradiation regardless of the time of EE breeding. The results show that serum leptin concentrations decreased in both the juvenile and adult EE groups compared to both the juvenile and adult SE groups, respectively, and these decreases of leptin level were detected after irradiation in both the juvenile and adult EE groups (**Figure 1**). We conclude that the suppressive effects of EE on leptin expression is maintained even after radiation exposure, regardless of the time of EE breeding.

### EE Improves Responsiveness to DNA Damage and Increases DNA Damage Repair Efficiency

**Figure 2:**
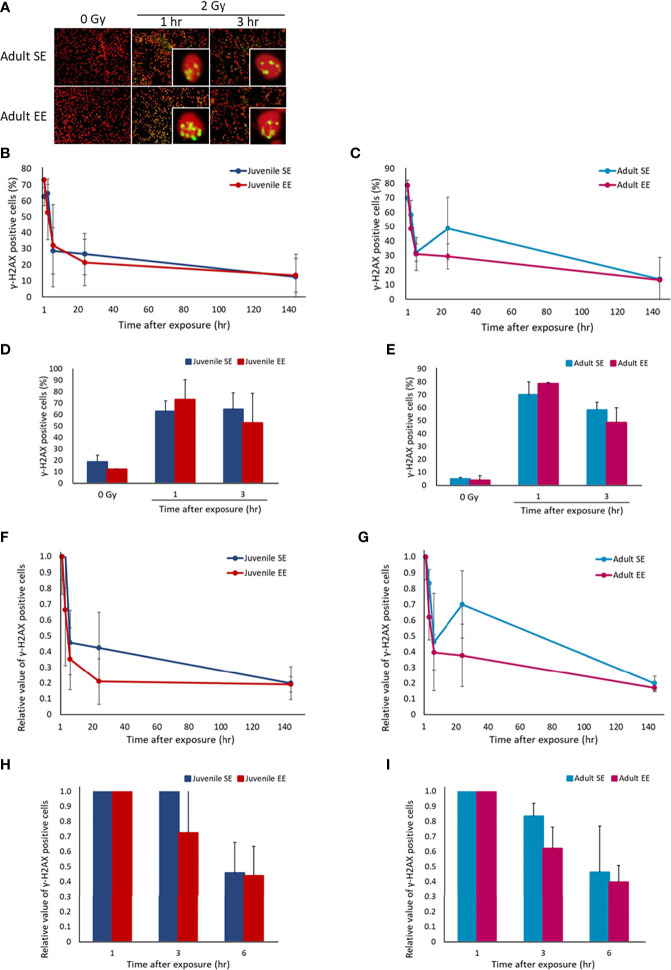
Effect of EE on DNA damage response. **(A)** Representative immunostaining image of γ-H2AX-positive cells. Green, γ-H2AX; Red, DNA stained by propidium iodide (400 x magnification). **(B, C)** Changes in γ-H2AX-positive cell rate over time after X-ray 2 Gy irradiation in lung tissue of juvenile SE/EE **(B)** or adult SE/EE **(C)** mice. Cells with 4 or more γ-H2AX foci per cell were defined as γ-H2AX-positive cells and at least 500 cells were randomly measured before calculating the ratio. The data show mean ± SEM; n = 3 mice per group. The statistical approach is Student’s t-test (two-tailed) (Juvenile group; 1-144 h after irradiation. Adult group; before irradiation and 3-144 h after irradiation) or Welch’s t-test (two-tailed) (Juvenile group; before irradiation. Adult group; 1 h after irradiation.) was performed according to the results of the F-test *via* the Microsoft Excel. **(D, E)** Changes in γ-H2AX-positive cell rate over time up to 3 h after X-ray 2 Gy irradiation in lung tissue of juvenile SE/EE **(D)** or adult SE/EE **(E)** mice. The data show mean ± SEM; n = 3 mice per group. The statistical approach is Student’s t-test (two-tailed) (Juvenile group; 1, 3 h after irradiation. Adult group; before irradiation and 3 h after irradiation) or Welch’s t-test (two-tailed) (Juvenile group; before irradiation. Adult group; 1 h after irradiation.) was performed according to the results of the F-test *via* the Microsoft Excel. **(F, G)** Changes in the rate of decrease in γ-H2AX-positive cells after X-ray 2 Gy irradiation in lung tissue of juvenile SE/EE **(F)** or adult SE/EE **(G)** mice. It is a relative value to the γ-H2AX-positive cell rate detected 1 h after irradiation. The data show mean ± SEM; n = 3 mice per group. The statistical approach is Student’s t-test (two-tailed) (Juvenile group; 1-144 h after irradiation. Adult group; 3-144 h after irradiation) or Welch’s t-test (two-tailed) (Adult group; 1 h after irradiation.) was performed according to the results of the F-test *via* the Microsoft Excel. **(H, I)** Changes in the rate of decrease in γ-H2AX-positive cells up to 6 h after irradiation with X-ray 2 Gy in lung tissue of juvenile SE/EE **(H)** or adult SE/EE **(I)** mice. The data show mean ± SEM; n = 3 mice per group. The statistical approach is Student’s t-test (two-tailed) (Juvenile group; 1-6 h after irradiation. Adult group; 3, 6 h after irradiation) or Welch’s t-test (two-tailed) (Adult group; 1 h after irradiation.) was performed according to the results of the F-test *via* the Microsoft Excel.

It has been reported that the incident of radiation-induced lung adenocarcinoma is high in B6C3F1 male mice strain (33). Therefore, the effect of EE on radiation-induced DNA damage response in the lung tissue was investigated by irradiating all experimental groups and collecting lung tissue over time. IHC was conducted using γ-H2AX, a marker of DNA DSBs, using the collected lung tissue. The results show that DNA damage levels peaked in all four experimental groups 1-3 h after irradiation, which confirmed the occurrence of a damage repair response (**Figures 2B, C**). When focusing on the initial response, the γ-H2AX-positive cell rate in both the juvenile and adult EE groups was higher than that in both the juvenile and adult SE groups at 1 h after irradiation though there were no statistical differences. At 3 h after irradiation, the γ-H2AX-positive cell rate in the both the juvenile and adult EE groups was clearly lower than that in both the juvenile and adult SE groups (**Figures 2D, E**). It was suggested that EE might rapidly activate the response to DNA damage and rapidly induced DNA damage repair mechanisms in the lung tissue regardless of the time of EE breeding. We also examined the effect of EE on the level of DNA damage before radiation exposure and found that the level was reduced in the both the juvenile and adult EE groups (**Figures 2D, E**). Similar experiments performed on liver tissue show that EE tended to improve the responsiveness to radiation-induced DNA damage and to reduce the level of spontaneous DNA damage as seen in lung tissue (**Supplementary Figures 1A–D**).

The effect of damage repair kinetics over time after radiation exposure was evaluated by calculating the relative DNA damage level with respect to the γ-H2AX-positive cell rate 1 h after irradiation and comparing the DNA damage repair efficiency. Interestingly, the γ-H2AX-positive cell reduction rate had a trend to higher in both the juvenile and adult EE groups than in both the juvenile and adult SE groups (**Figures 2F** **–I**). These results suggest that radiation-induced DNA damage was repaired more efficiently in the lung tissue owing to EE. Similar experiments performed on liver tissue show that significant increases in DNA damage repair efficiency sustain from 1 to 144 h after irradiation was observed in the juvenile EE group as seen in lung tissue. In contrast, the repair efficiency of liver tissue in the adult EE group was increased only at 3 and 144 h after irradiation compared with that in the adult SE group, suggesting that the effect on DNA damage repair efficiency in liver tissue may depend on the time of EE breading (**Supplementary Figures 1E–H**).

### EE Improves the Responsiveness of Activated Macrophages and Rapidly Reduces Inflammation

**Figure 3:**
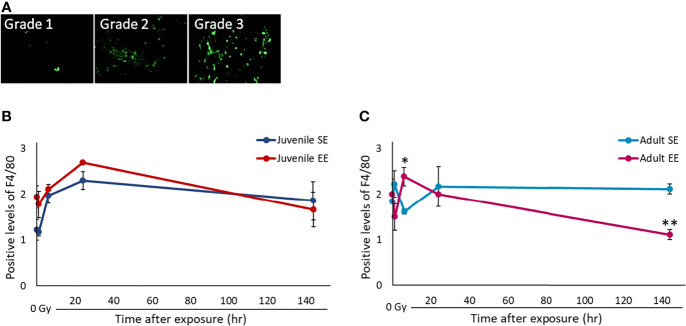
Effect of EE on inflammatory response by activated macrophages. **(A)** Representative images of F4/80 immunostaining. F4/80 positive intensity grades were 1, minimal; 2, mild; 3, moderate. Green: F4/80 (400 x magnification). As the background level of F4/80 staining was high in lung tissue, measurement of F4/80 positivity based on signal intensity was impossible as in liver tissue (**Supplementary Figure 2**). Therefore, we validated the F4/80 positivity by scoring based on the staining pattern in the lung. **(B, C)** Changes in F4/80-positivity over time after X-ray non-irradiation and 2 Gy irradiation in lung tissue of juvenile SE/EE **(B)** or adult SE/EE **(C)** mice. The data show mean ± SEM; n = 3 mice per group. The statistical approach is Student’s t-test (two-tailed) (Juvenile group; before irradiation and 1, 6, 144 h after irradiation. Adult group; before irradiation and 1, 6, 144 h after irradiation) or Welch’s t-test (two-tailed) (Juvenile group; 24 h after irradiation. Adult group; 24 h after irradiation.) was performed according to the results of the F-test *via* the Microsoft Excel. *P < 0.05, **P < 0.01 compared to the SE group.

Changes in the tissue microenvironment, represented by the inflammatory reaction of tissues, play an important role in the carcinogenic process. The effect of EE on the tissue inflammatory reaction after radiation exposure was investigated by irradiating all experimental groups with 2 Gy and collecting the lung tissue over time. IHC was performed using the F4/80 protein, a marker of activated macrophages, using the collected lung tissue (**Figure 3A**). First, we checked the effect of basal immunity. The results show that the expression level of F4/80-positive cells in both the juvenile and adult EE groups without radiation exposure was higher than those in both the juvenile and adult SE groups, suggesting that EE improves basal immunity regardless of the timing of EE. Next, the expression level of F4/80-positive cells after radiation was evaluated. The expression level of F4/80-positive cells in both the juvenile and adult EE groups was highest after 6-24 h of irradiation. The expression level of the positive cells in the EE group at the peak, i.e., 24 h after irradiation in the juvenile EE group and 6 h after irradiation in the adult EE group, was statistically higher in both the juvenile and adult EE groups than in both the juvenile and adult SE groups (6 h after irradiation in the adult group; *P* < 0.05) (**Figures 3B, C**). These results suggest that the inflammatory response was activated by radiation, and the responsiveness of the activated macrophages was enhanced by EE. Expression levels in both the juvenile and adult EE groups tended to return to pre-irradiation levels at 144 h after irradiation. In contrast, macrophage activation in both the juvenile and adult SE groups, which increased 24 h after irradiation, persisted after 144 h and did not return to pre-irradiation levels (**Figures 3B, C**). These results suggest that both the juvenile and adult SE groups induced a prolonged inflammatory reaction, which may lead to chronic inflammation, whereas both the juvenile and adult EE groups showed a slightly rapid and efficient reduction of the radiation-induced inflammatory reaction.

Similar experiments performed on liver tissue showed that, as seen in lung tissue, EE improved the responsiveness of activated macrophages and tended to reduce inflammation 144 h after irradiation compared to both the juvenile and adult SE groups. It was also confirmed that juvenile EE increased the expression levels of activated macrophages when not irradiated (**Supplementary Figure 2**).

### EE Controls M1/M2 Polarization of Macrophages and Processes the Inflammatory Reaction After Radiation Exposure as Acute Inflammation

**Figure 4:**
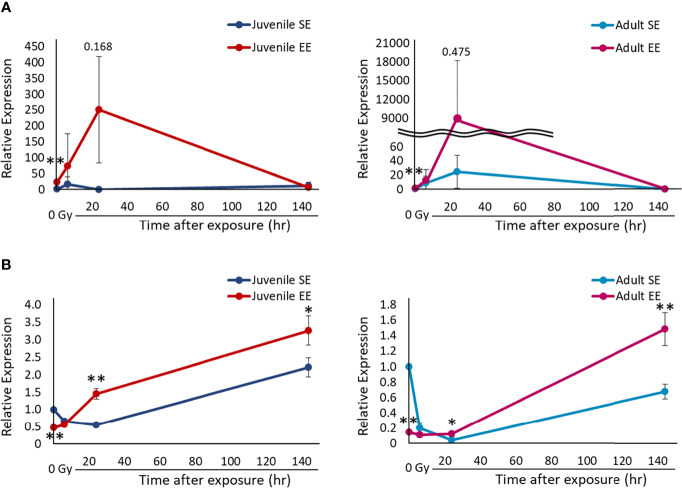
Effect of EE on the polarization of macrophages. **(A)** Changes in *TNF* gene expression over time after X-ray non-irradiation and 2 Gy irradiation in lung tissue of juvenile SE/EE (left) or adult SE/EE (right) mice. Gene expression was normalized based on *GAPDH* expression. It is shown as a relative value to gene expression in non-irradiation SE mice. The data show mean ± SD; n = 3 independent experiments. The statistical approach is Student’s t-test (two-tailed) (Juvenile group; before irradiation and 1, 6, 144 h after irradiation. Adult group; before irradiation and 1, 6, 144 h after irradiation) or Welch’s t-test (two-tailed) (Juvenile group; 24 h after irradiation. Adult group; 24 h after irradiation.) was performed according to the results of the F-test *via* the Microsoft Excel. *P < 0.05, **P < 0.01 compared to the SE group. **(B)** Changes in *Arg1* gene expression over time after X-ray non-irradiation and 2 Gy irradiation in lung tissue of juvenile SE/EE (left) or adult SE/EE (right) mice. Gene expression was normalized based on *GAPDH* expression. It is shown as a relative value to gene expression in non-irradiation SE mice. The data show mean ± SD; n = 3 independent experiments. The statistical approach is Student’s t-test (two-tailed) (Juvenile group; before irradiation and 1-144 h after irradiation. Adult group; before irradiation and 1-144 h after irradiation) was performed according to the results of the F-test *via* the Microsoft Excel. *P < 0.05, **P < 0.01 compared to the SE group.

Evaluation of the macrophage sub-population in the inflammatory reaction is important to assess the effect of EE on the carcinogenesis process. Therefore, we decided to investigate whether the modulation of M1/M2 polarization of macrophages by EE is involved in the mechanism by which EE affects the inflammatory reaction. All experimental groups were irradiated, and lung tissue was collected over time. The collected lung tissue was used to set the target genes as *TNF*, which is specifically expressed by M1 macrophages, and *Arg1*, which is specifically expressed by M2 macrophages. Their expression levels were quantified by RT-qPCR. *TNF* expression before radiation exposure was statistically higher in both the juvenile and adult EE groups than in both the juvenile and adult SE groups in qPCR analysis (*P* < 0.01) (**Figure 4A**), which is consistent with the increased expression levels of activated macrophages seen in both the juvenile and adult EE groups before radiation exposure in IHC analysis (**Figure 3**). These results suggest that EE increases the expression of pro-inflammatory M1 macrophages and improves basal immunity. *TNF* expression level was strikingly higher in both the juvenile and adult EE groups than in both the juvenile and adult SE groups up to 24 h after irradiation. Meanwhile, at 144 h after irradiation, *TNF* expression level was lower in the juvenile EE group than in the juvenile SE group. For the adult EE group, *TNF* expression level at 144 h after irradiation was higher than that of the adult SE group (relative expression level 144 h after irradiation; adult SE, 0.22: adult EE, 0.53). However, the reduction rate in *TNF* expression level between 24 and 144 h after irradiation was markedly higher in both the juvenile and adult EE groups than in both the juvenile and adult SE groups (**Figure 4A**). These results suggest that EE increases inflammatory responsiveness by markedly increasing *TNF* expression by 24 h after irradiation, and then contributes to the reducing inflammation by rapidly decreasing *TNF* expression level at 144 h after irradiation.

In contrast, *Arg1* expression levels were statistically lower in both the juvenile and adult EE groups than in both the juvenile and adult SE groups before and up to 6 h after irradiation (before irradiation; *P* < 0.01). However, both the juvenile and adult EE groups exhibited statistically higher *Arg1* expression than both the juvenile and adult SE groups 24 h after irradiation and onwards (*P* < 0.01 or 0.05) (**Figure 4B**). These results indicate that EE modulates M1/M2 polarization in macrophages, suggesting that switching between M1/M2 polarization by EE may occur 24 h after irradiation. In other words, EE promotes M1 polarization of macrophages and induce an active inflammatory reaction up to 6 h after irradiation, with an anti-inflammatory reaction activated by promoting M2 polarization 24 h after irradiation and onwards.

The inflammatory reaction could be processed as an acute inflammation which is marked by rapid switching of M1/M2 polarization in both the juvenile and adult EE groups, whereas chronic inflammation which may have been induced in the juvenile and adult SE groups. These results support the data shown in **Figure 3**. Similar experiments performed in liver tissue showed that, as seen in lung tissue, EE affected macrophage polarization and tended to produce an active anti-inflammatory reaction (data not shown).

## Discussion

Many cancers interact with the endocrine system and appear to have an especially strong relationship with obesity. There has been increasing attention to the relationship between cancer and leptin, which is abundant in adipose tissue (34–36). In fact, increased serum leptin levels have been reported to increase the risk of developing several cancers, including melanoma and colon cancer (37–40). Meanwhile, EE induces BDNF expression in the hypothalamus and activates sympathetic nerves to change adipocytes from white to brown, which contributes to fat loss (41). EE is also known to reduce leptin secreted from white adipose tissue, thereby suppressing tumor growth (13, 15). However, previous EE-related studies often initiated EE breeding at 3 weeks of age immediately after weaning and did not consider the effect of the timing of manipulating the breeding environment on the EE effects. Consistent with previous studies, our current study clearly shows that steady-state serum leptin levels were reduced in EE mice, suggesting that EE reduces the risk of carcinogenesis by suppressing leptin secretion. This EE effect was observed regardless of the duration of EE breeding. We also found that the serum leptin levels in EE mice decreased even after radiation exposure, and we anticipated that the inhibitory effect of EE on tumor growth would continue even after radiation exposure (**Figure 1**). The results confirmed that these EE effects appeared regardless of the time when the breeding environment was manipulated. Even adult mice with an established immune system may be able to reduce carcinogenesis risk by performing EE before radiation exposure.

Although many reports have shown that EE is effective in models of pancreatic cancer (42, 43), neuroglioma (44), and lung cancer (42), the effect on radiation-induced carcinogenic processes is unknown and needs to be analyzed. However, the carcinogenic process resulting from radiation exposure progresses while various cell and tissue responses, such as DNA damage repair and inflammatory reaction, are activated in a complex manner, and thus evaluating carcinogenic risk with a one-sided analysis is difficult. In other words, a correct understanding of radiation-induced carcinogenesis is necessary when investigating whether EE is effective against this disease. To that end, the response of cells, tissues, organs, and individuals following radiation exposure must be investigated by focusing on various molecular markers and basic data that are analyzed over time. Therefore, we evaluated the changes in γ-H2AX levels and inflammatory reaction levels in radiation-exposed mice.

This study revealed that the lung tissue of EE mice responded to radiation-induced DNA damage with high sensitivity and accuracy. In addition, increased DNA damage repair efficiency was also confirmed in the lung tissue of EE mice, regardless of the time when the breeding environment was manipulated (**Figure 2**). In the liver tissue, juvenile EE was shown to increase DNA damage repair efficiency (**Supplementary Figure 1**). These results suggest that EE maintains genome stability by activating the DNA damage response, which might result in a reduction in the number of cells that become cancerous and contribute to reducing carcinogenesis risk.

The same SE/EE mouse-derived serum was added to cultured cells, and the DNA damage repair process after irradiation was monitored, and the results confirmed that the damage repair efficiency was increased in cells supplemented with serum derived from EE mice (unpublished data). There may be a component in the serum of EE mice that increases the rate of DNA damage repair. However, the identity of the components that enhance DNA damage responsiveness remains unclear.

In this study, in the lung and liver tissues of EE mice showed reduced levels of DNA damage pre-irradiation (**Figure 2** and **Supplementary Figure 1**), suggesting that 8 weeks of EE breeding lowered the baseline levels of DNA damage. One of the reasons for the lower baseline is the rapid damage repair response. In our study, it was shown that EE increased the efficiency of DNA damage repair after irradiation, but it was also thought that EE contributed to the improvement of damage repair efficiency before irradiation.

Our results suggest that EE may be useful as a measure for reducing carcinogenesis risk in the DNA damage repair reaction, which is one of the initial responses after radiation exposure. We therefore, also focused on the inflammatory response. First, we showed that EE increased the expression of activated macrophages at rest and significantly increased *TNF* expression specifically produced by M1 macrophages (**Figures 3**, **4**). These results suggest that EE enhances basal immunity and enhances responsiveness to external stimuli, such as radiation exposure. Other researchers have shown that EE upgrades immune functions such as improvements in NK cell activity (42, 44, 45) and macrophage phagocytosis (31, 46); our results support these findings.

Next, analysis of changes in the inflammatory reaction after radiation exposure shows a rapid increase in the expression of activated macrophages in EE mice, and it was confirmed that the peak expression level of macrophages after irradiation was higher than that in SE mice (**Figure 3**). In other words, EE was expected to increase the number of activated macrophages drawn to the damaged area after radiation induces an inflammatory reaction, and it is thought that EE has a function of enhancing immune actions. Studies on the effect of EE on the immune response show that EE significantly increases macrophage influx into the abdominal cavity 24 h after inflammatory stimulation (46). These results are consistent with those of this study, in which EE increased the expression of activated macrophages from 6 to 24 h after irradiation.

RT-qPCR analysis for validation of M1/M2 polarization showed that the expression of *TNF*, an M1 macrophage marker, was high in EE mice up to 24 h after irradiation, whereas the expression of *Arg1*, an M2 macrophage marker, was reduced in EE mice up to 6 h after irradiation (**Figure 4**). It was thought that M1 polarization of macrophages was accelerated in EE mice up to 24 h after irradiation, and that this contributed to the activation of inflammation. The results reported by the Kobayashi group (31), which showed that EE increases the expression of macrophages with M1-type traits as well as chemokine production, also support our discussion.

M1 macrophages may promote phagocytosis and inflammatory reactions. At the same time, they release nitric oxide and reactive oxygen species, which may damage neighboring tissues (27–30). In fact, control of M1/M2 polarization is important in many diseases, and chronic M1 polarization is known to be correlated with cardiovascular disease (47), progression of obesity, and the development of diabetes (48, 49). Therefore, inducing the expression of M2 macrophages, which have anti-inflammatory actions, is thought to be important for reducing and terminating the excessive damage reaction resulting from long-term M1 polarization. Supporting these points is the fact that the anti-inflammatory reaction was induced by an increase in *Arg1* expression in SE mice 144 h after irradiation and in EE mice 24 h after irradiation (**Figure 4**). Rapid *Arg1* induction in EE mice suggests that EE is a rapidly reducing inflammation activated by M1 macrophages.

IHC analysis revealed that radiation-induced inflammation resolved in EE mice 144 h after irradiation, whereas inflammation persisted in SE mice even 144 h after irradiation (**Figure 3**). Chronic inflammation owing to residual activated macrophages leads to an increased risk of carcinogenesis (20). Based on these results, we conclude that the inflammation reaction persisted in SE mice, whereas EE reduced inflammation and suppressed the formation of a cancer-promoting microenvironment.

Analysis of gene expression over time shows that switching of macrophage M1/M2 polarization in mouse lung tissue was likely to occur 6–24 h after radiation exposure. At this time point the expression of activated macrophages reached its peak (**Figure 3**) and DNA damage was repaired, which is marked by the disappearance of γ-H2AX foci (**Figure 2**). We have previously shown that B6C3F1 mice exposed to 3.8–4 Gy have increased number of γ-H2AX-positive cells in the lungs and liver immediately after irradiation, returning to pre-irradiation levels 24 h after irradiation (unpublished data). It can be inferred from these results that some tissue-level events occurred within 24 h after irradiation. During an inflammatory response, glycolysis is enhanced and M1 macrophages are increased 1–6 h after stimulation, whereas fatty acid metabolism was increased and M2 macrophages are increased 12–24 h after stimulation (50). It is thought that there were large changes in macrophage phenotype and cell metabolism 6–24 h after stimulation.

Studies of the thymic gland after 1 Gy of X-ray irradiation in juvenile mice revealed that apoptotic cells dramatically disappear 10.5–12 h after irradiation (51). Radiation-induced apoptosis in the spinal cord of rats, regardless of age, occurred frequently 8 h after irradiation, returning to pre-irradiation levels 24 h after irradiation (52). These reports also suggest that apoptosis may occur within 24 h of irradiation. In fact, we measured apoptotic-like cells by the pan-γ-H2AX staining pattern (18). Our preliminary data suggests an increase of apoptotic cells in 3–24 h after irradiation in all four experimental groups (data not shown). In this study, we found that activated macrophage expression peaked at 24 h after irradiation (**Figure 3**). Based on these results, apoptosis may have been induced when the living body was stimulated by radiation, but it is possible that these apoptotic cells underwent phagocytosis by macrophages, leading to further induction of inflammatory reactions. In other words, it is thought that the timing at which phagocytic macrophages were attracted after apoptosis coincided with the peak of the inflammatory reaction.

We hypothesized that DNA damage repair occurs as a result of radiation exposure, and cells that cannot be repaired are handled by apoptosis. Macrophages that phagocytose apoptotic cells are attracted to the damage site while exhibiting M1-type traits, activating the inflammatory response. This occurs within 24 h after irradiation, after which the anti-inflammatory reaction and tissue repair occur. EE is suggested to control the transition to chronic inflammation and to exhibit anti-carcinogenic effects by efficiently promoting the switching of this M1/M2 polarization. Although the tissue response between 6 h and 24 h after irradiation was not analyzed, this hypothesis is strongly supported by results from other studies (31, 51).

In conclusion, this study examined the usefulness of EE as a measure to reduce the risk of carcinogenesis after radiation exposure. The results confirmed that the inhibitory effect of EE on tumor growth was maintained even after radiation exposure owing to EE breeding (i.e., improved living environment) before radiation exposure. EE was confirmed to improve responsiveness to radiation-induced DNA damage and basal immunity, further suppressing the chronic inflammatory response. These EE effects were observed regardless of the time at which the breeding environment was manipulated. In other words, the health-promoting effects of EE even after entering the adult period were clarified. This study revealed that EE could change the initial response after radiation exposure, and considering these results collectively, EE might lead to a reduction of the risk of radiation-induced carcinogenesis.

## Data Availability Statement

The raw data supporting the conclusions of this article will be made available by the authors, without undue reservation.

## Ethics Statement

Animal care and experimental schedules were approved by the National Institute of Radiological Sciences (NIRS), National Institutes for Quantum and Radiological Science and Technology (QST) of Japan and were in strict accordance with the guidelines of the Institute.

## Author Contributions

SS, MN, TM, SK, YS, and AJN conceived and planned the experiments. Data were collected by SS, KK, MM, TO, SM, SO, SY, MN, and TM. Analysis was carried out by SS, KK, MM, TO, SM, SO, and AJN. SS, and AJN wrote the manuscript. AJN take final responsibility for this article. All authors have read and agreed to the published version of the manuscript.

## Funding

This work was supported by Ministry of Education, Culture, Sports, Science and Technology of Japan *via* the Nuclear Energy Science and Technology and Human Resource Development Project through Concentrating Wisdom to TM, SK, YS, and AJN.

## Conflict of Interest

The authors declare that the research was conducted in the absence of any commercial or financial relationships that could be construed as a potential conflict of interest.

## Publisher’s Note

All claims expressed in this article are solely those of the authors and do not necessarily represent those of their affiliated organizations, or those of the publisher, the editors and the reviewers. Any product that may be evaluated in this article, or claim that may be made by its manufacturer, is not guaranteed or endorsed by the publisher.

## References

[1] Honda S Shibata Y Mine M Imamura Y Tagawa M Nakane Y. Mental Health Conditions Among Atomic Bomb Survivors in Nagasaki. Psychiatry Clin Neurosci (2002) 56:575–83. doi: 10.1046/j.1440-1819.2002.01057.x 12193250

[2] Patel ZS Brunstetter TJ Tarver WJ Whitmire AM Zwart SR Smith SM. Red Risks for a Journey to the Red Planet: The Highest Priority Human Health Risks for a Mission to Mars. NPJ Microgravity (2020) 6:33. doi: 10.1038/s41526-020-00124-6 33298950 PMC7645687

[3] Hori H Kim Y. Inflammation and Post-Traumatic Stress Disorder. Psychiatry Clin Neurosci (2019) 73:143–53. doi: 10.1111/pcn.12820 30653780

[4] Ma X Aoki T Tsuruyama T Narumiya S. Definition of Prostaglandin E2-EP2 Signals in the Colon Tumor Microenvironment That Amplify Inflammation and Tumor Growth. Cancer Res (2015) 75:2822–32. doi: 10.1158/0008-5472.CAN-15-0125 26018088

[5] Umamaheswaran S Dasari SK Yang P Lutgendorf SK Sood AK. Stress, Inflammation, and Eicosanoids: An Emerging Perspective. Cancer Metastasis Rev (2018) 37:203–11. doi: 10.1007/s10555-018-9741-1 PMC6237279 29948328

[6] Song H Saito E Sawada N Abe SK Hidaka A Shimazu T. Perceived Stress Level and Risk of Cancer Incidence in a Japanese Population: The Japan Public Health Center (JPHC)-Based Prospective Study. Sci Rep (2017) 7:12964. doi: 10.1038/s41598-017-13362-8 29021585 PMC5636815

[7] Slater AM Cao L. A Protocol for Housing Mice in an Enriched Environment. J Vis Exp (2015) 2015:e52874. doi: 10.3791/52874 PMC4544994 26131694

[8] van Praag H Kempermann G Gage FH. Neural Consequences of Enviromental Enrichment. Nat Rev Neurosci (2000) 1:191–8. doi: 10.1038/35044558 11257907

[9] Cao L Jiao X Zuzga DS Liu Y Fong DM Young D. VEGF Links Hippocampal Activity With Neurogenesis, Learning and Memory. Nat Genet (2004) 36:827–35. doi: 10.1038/ng1395 15258583

[10] McMurphy T Huang W Queen NJ Ali S Widstrom KJ Liu X. Implementation of Environmental Enrichment After Middle Age Promotes Healthy Aging. Aging (Albany NY) (2018) 10:1698–721. doi: 10.18632/aging.101502 PMC6075449 30036185

[11] Wu Y Gan Y Yuan H Wang Q Fan Y Li G. Enriched Environment Housing Enhances the Sensitivity of Mouse Pancreatic Cancer to Chemotherapeutic Agents. Biochem Biophys Res Commun (2016) 473:593–9. doi: 10.1016/j.bbrc.2016.03.128 27033603

[12] Cao L Choi EY Liu X Martin A Wang C Xu X. White to Brown Fat Phenotypic Switch Induced by Genetic and Environmental Activation of a Hypothalamic-Adipocyte Axis. Cell Metab (2011) 14:324–38. doi: 10.1016/j.cmet.2011.06.020 PMC3172615 21907139

[13] Cao L During MJ. What Is the Brain-Cancer Connection? Annu Rev Neurosci (2012) 35:331–45. doi: 10.1146/annurev-neuro-062111-150546 22462541

[14] Shtoots L Richter-Levin G Hugeri O Anunu R. Juvenile Stress Leads to Long-Term Immunological Metaplasticity-Like Effects on Inflammatory Responses in Adulthood. Neurobiol Learn Mem (2018) 154:12–21. doi: 10.1016/j.nlm.2017.09.008 28962838

[15] Cao L Liu X Lin EJD Wang C Choi EY Riban V. Environmental and Genetic Activation of a Brain-Adipocyte BDNF/Leptin Axis Causes Cancer Remission and Inhibition. Cell (2010) 142:52–64. doi: 10.1016/j.cell.2010.05.029 20603014 PMC3784009

[16] Symington LS. DNA Repair: Making the Cut. Nature (2014) 514:39–40. doi: 10.1038/nature13751 25231858

[17] Rogakou EP Pilch DR Orr AH Ivanova VS Bonner WM. DNA Double-Stranded Breaks Induce Histone H2AX Phosphorylation on Serine 139. J Biol Chem (1998) 273:5858–68. doi: 10.1074/jbc.273.10.5858 9488723

[18] Bonner WM Redon CE Dickey JS Nakamura AJ Sedelnikova OA Solier S. γh2ax and Cancer. Nat Rev Cancer (2008) 8:957–67. doi: 10.1038/nrc2523 PMC3094856 19005492

[19] Shapouri-Moghaddam A Mohammadian S Vazini H Taghadosi M Esmaeili SA Mardani F. Macrophage Plasticity, Polarization, and Function in Health and Disease. J Cell Physiol (2018) 233:6425–40. doi: 10.1002/jcp.26429 PMC13482560 29319160

[20] Chen L Deng H Cui H Fang J Zuo Z Deng J. Inflammatory Responses and Inflammation-Associated Diseases in Organs. Oncotarget (2018) 9:7204–18. doi: 10.18632/oncotarget.23208 PMC5805548 29467962

[21] Chen X Tang J Shuai W Meng J Feng J Han Z. Macrophage Polarization and Its Role in the Pathogenesis of Acute Lung Injury/Acute Respiratory Distress Syndrome. Inflamm Res (2020) 69:883–95. doi: 10.1007/s00011-020-01378-2 PMC7347666 32647933

[22] Herold S Mayer K Lohmeyer J. Acute Lung Injury: How Macrophages Orchestrate Resolution of Inflammation and Tissue Repair. Front Immunol (2011) 2:65. doi: 10.3389/fimmu.2011.00065 22566854 PMC3342347

[23] Orecchioni M Ghosheh Y Pramod AB Ley K. Macrophage Polarization: Different Gene Signatures in M1(Lps+) *vs*. Classically and M2(LPS-) *vs*. Alternatively Activated Macrophages. Front Immunol (2019) 10:1084. doi: 10.3389/fimmu.2019.01084 31178859 PMC6543837

[24] Wu J Zhang L Shi J He R Yang W Habtezion A. Macrophage Phenotypic Switch Orchestrates the Inflammation and Repair/Regeneration Following Acute Pancreatitis Injury. EBioMedicine (2020) 58:102920. doi: 10.1016/j.ebiom.2020.102920 32739869 PMC7399125

[25] Shacter E Weitzman SA. Chronic Inflammation and Cancer. Oncol (Williston Park) (2002) 16:217–26. 11866137

[26] Mège JL Mehraj V Capo C. Macrophage Polarization and Bacterial Infections. Curr Opin Infect Dis (2011) 24:230–4. doi: 10.1097/QCO.0b013e328344b73e 21311324

[27] Murray PJ Wynn TA. Protective and Pathogenic Functions of Macrophage Subsets. Nat Rev Immunol (2011) 11:723–37. doi: 10.1038/nri3073 PMC3422549 21997792

[28] Sica A Mantovani A. Macrophage Plasticity and Polarization: *In Vivo* Veritas. J Clin Invest (2012) 122:787–95. doi: 10.1172/JCI59643 PMC3287223 22378047

[29] Oishi Y Manabe I. Macrophages in Inflammation, Repair and Regeneration. Int Immunol (2018) 30:511–528. doi: 10.1093/intimm/dxy054 30165385

[30] Hamidzadeh K Christensen SM Dalby E Chandrasekaran P Mosser DM. Macrophages and the Recovery From Acute and Chronic Inflammation. Annu Rev Physiol (2017) 79:567–592. doi: 10.1146/annurev-physiol-022516-034348 27959619 PMC5912892

[31] Otaki M Hirano T Yamaguchi Y Kaida K Koshika S Nagata K. Changes in the Function and Phenotype of Resident Peritoneal Macrophages After Housing in an Enriched Environment. Int Immunopharmacol (2018) 65:44–49. doi: 10.1016/j.intimp.2018.09.037 30273916

[32] Morioka T Blyth BJ Imaoka T Nishimura M Takeshita H Shimomura T. Establishing the Japan-Store House of Animal Radiobiology Experiments (J-SHARE), A Large-Scale Necropsy and Histopathology Archive Providing International Access to Important Radiobiology Data. Int J Radiat Biol (2019) 95:1372–7. doi: 10.1080/09553002.2019.1625458 31145030

[33] Shang Y Kakinuma S Yamauchi K Morioka T Kokubo T Tani S. Cancer Prevention by Adult-Onset Calorie Restriction After Infant Exposure to Ionizing Radiation in B6C3F1 Male Mice. Int J Cancer (2014) 135:1038–47. doi: 10.1002/ijc.28751 24482070

[34] Hassan QN Queen NJ Cao L. Regulation of Aging and Cancer by Enhanced Environmental Activation of a Hypothalamic-Sympathoneural-Adipocyte Axis. Transl Cancer Res (2020) 9:5687–99. doi: 10.21037/tcr.2020.02.39 PMC7595574 33134111

[35] Deng T Lyon CJ Bergin S Caligiuri MA Hsueh WA. Obesity, Inflammation, and Cancer. Annu Rev Pathol Mech Dis (2016) 11:421–49. doi: 10.1146/annurev-pathol-012615-044359 27193454

[36] De Pergola G Silvestris F. Obesity as a Major Risk Factor for Cancer. J Obes (2013) 2013:291546. doi: 10.1155/2013/291546 24073332 PMC3773450

[37] Gogas H Trakatelli M Dessypris N Terzidis A Katsambas A Chrousos GP. Melanoma Risk in Association With Serum Leptin Levels and Lifestyle Parameters: A Case-Control Study. Ann Oncol (2008) 19:384–9. doi: 10.1093/annonc/mdm464 17925285

[38] Nachat-Kappes R Pinel A Combe K Lamas B Farges MC Rossary A. Effects of Enriched Environment on COX-2, Leptin and Eicosanoids in a Mouse Model of Breast Cancer. PloS One (2012) 7:e51525. doi: 10.1371/journal.pone.0051525 23272114 PMC3521763

[39] Garofalo C Koda M Cascio S Sulkowska M Kanczuga-Koda L Golaszewska J. Increased Expression of Leptin and the Leptin Receptor as a Marker of Breast Cancer Progression: Possible Role of Obesity-Related Stimuli. Clin Cancer Res (2006) 12:1447–53. doi: 10.1158/1078-0432.CCR-05-1913 16533767

[40] Ishikawa M Kitayama J Nagawa H. Enhanced Expression of Leptin and Leptin Receptor (OB-R) in Human Breast Cancer. Clin Cancer Res (2004) 10:4325–31. doi: 10.1158/1078-0432.CCR-03-0749 15240518

[41] During MJ Liu X Huang W Magee D Slater A McMurphy T. Adipose VEGF Links the White-to-Brown Fat Switch With Environmental, Genetic, and Pharmacological Stimuli in Male Mice. Endocrinology (2015) 156:2059–73. doi: 10.1210/en.2014-1905 PMC4430610 25763639

[42] Song Y Gan Y Wang Q Meng Z Li G Shen Y. Enriching the Housing Environment for Mice Enhances Their NK Cell Antitumor Immunity *via* Sympathetic Nerve-Dependent Regulation of NKG2D and CCR5. Cancer Res (2017) 77:1611–22. doi: 10.1158/0008-5472.CAN-16-2143 28082402

[43] Li G Gan Y Fan Y Wu Y Lin H Song Y. Enriched Environment Inhibits Mouse Pancreatic Cancer Growth and Down-Regulates the Expression of Mitochondria-Related Genes in Cancer Cells. Sci Rep (2015) 5:7856. doi: 10.1038/srep07856 PMC4297951 25598223

[44] Garofalo S D’Alessandro G Chece G Brau F Maggi L Rosa A. Enriched Environment Reduces Glioma Growth Through Immune and Non-Immune Mechanisms in Mice. Nat Commun (2015) 6:6623. doi: 10.1038/ncomms7623 25818172 PMC4389244

[45] Benaroya-Milshtein N Hollander N Apter A Kukulansky T Raz N Wilf A. Environmental Enrichment in Mice Decreases Anxiety, Attenuates Stress Responses and Enhances Natural Killer Cell Activity. Eur J Neurosci (2004) 20:1341–7. doi: 10.1111/j.1460-9568.2004.03587.x 15341605

[46] Brod S Gobbetti T Gittens B Ono M Perretti M D’Acquisto F. The Impact of Environmental Enrichment on the Murine Inflammatory Immune Response. JCI Insight (2017) 2:e90723. doi: 10.1172/jci.insight.90723 28405616 PMC5374068

[47] Kirbiš S Breskvar UD Šabovič M Zupan I Sinkovič A. Inflammation Markers in Patients With Coronary Artery Disease - Comparison of Intracoronary and Systemic Levels. Wien Klin Wochenschr (2010) 122:31–4. doi: 10.1007/s00508-010-1343-z 20517668

[48] Odegaard JI Chawla A. Mechanisms of Macrophage Activation in Obesity-Induced Insulin Resistance. Nat Clin Pract Endocrinol Metab (2008) 4:619–26. doi: 10.1038/ncpendmet0976 PMC3381907 18838972

[49] Herder C Dalmas E Böni-Schnetzler M Donath MY. The IL-1 Pathway in Type 2 Diabetes and Cardiovascular Complications. Trends Endocrinol Metab (2015) 26:551–63. doi: 10.1016/j.tem.2015.08.001 26412156

[50] Oishi Y Spann NJ Link VM Muse ED Strid T Edillor C. SREBP1 Contributes to Resolution of Pro-Inflammatory TLR4 Signaling by Reprogramming Fatty Acid Metabolism. Cell Metab (2017) 25:412–27. doi: 10.1016/j.cmet.2016.11.009 PMC5568699 28041958

[51] Iyoda T Nagata K Akashi M Kobayashi Y. Neutrophils Accelerate Macrophage-Mediated Digestion of Apoptotic Cells *In Vivo* as Well as *In Vitro* . J Immunol (2005) 175:3475–83. doi: 10.4049/jimmunol.175.6.3475 16148089

[52] Li YQ Wong CS. Radiation-Induced Apoptosis in the Neonatal and Adult Rat Spinal Cord. Radiat Res (2000) 154:268–76. doi: 10.1667/0033-7587(2000)154[0268:RIAITN]2.0.CO;2 10956432

