# Supplementary material for: An Enriched Environment Alters DNA Repair and Inflammatory Responses After Radiation Exposure
Source: Front Immunol. 2021 Oct 22;12:760322. doi: 10.3389/fimmu.2021.760322 (PMC8570081; doi:10.3389/fimmu.2021.760322)
Supplement: Supplementary file 1 [file DataSheet_1.docx]

Supplementary Material


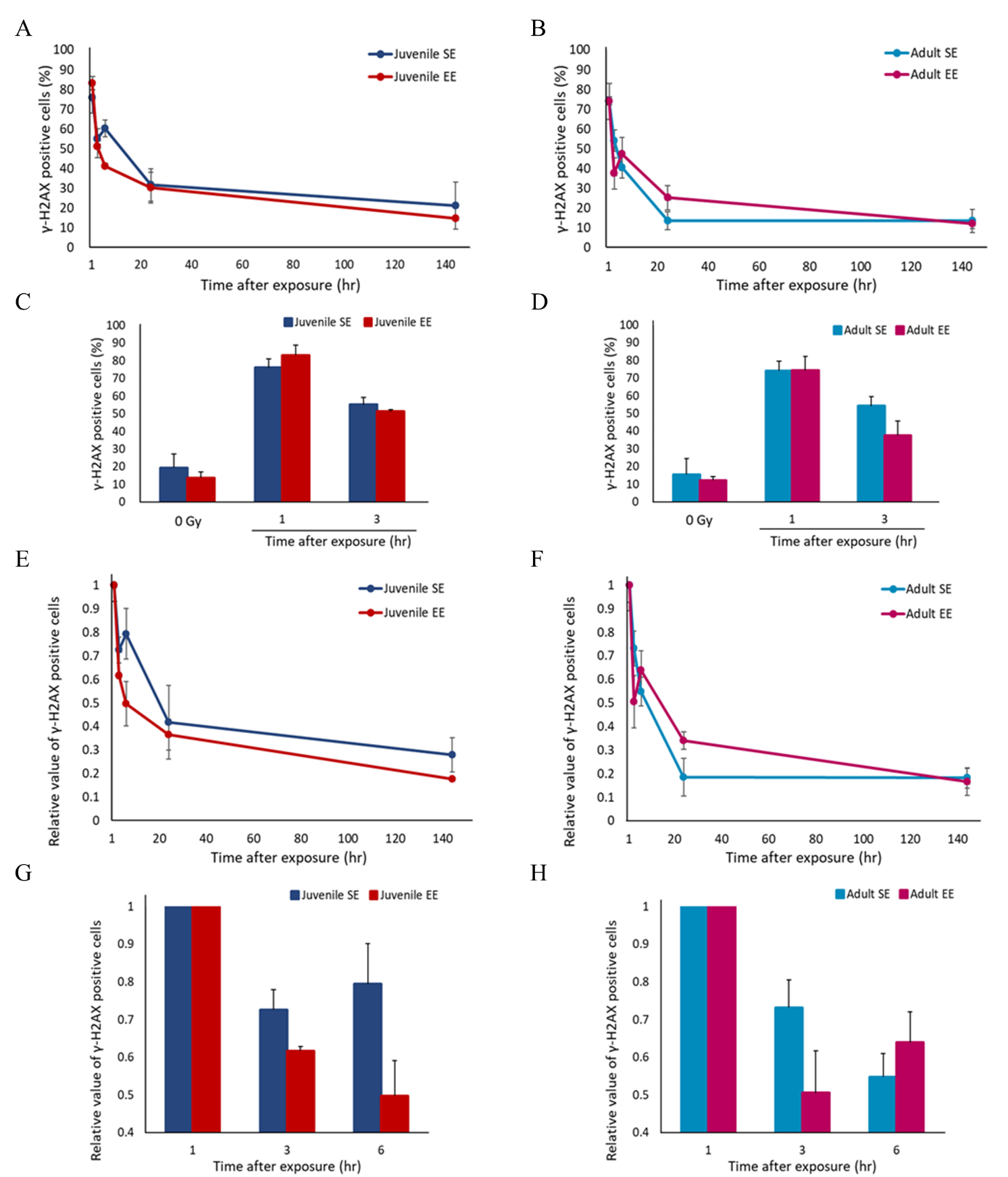


## Supplementary Figure 1. Effect of EE on DNA damage response in liver tissue.

(A, B) Changes in γ-H2AX-positive cell rate over time after X-ray 2 Gy irradiation in liver tissue of juvenile SE / EE (A) or adult SE / EE (B) mice. Cells with 4 or more γ-H2AX foci per cell were defined as γ-H2AX-positive cells and at least 500 cells were randomly measured. The data show mean ± SEM; n = 3 mice per group. The statistical approach is Student's t-test (two-tailed) (Juvenile group; before irradiation and 1-24 h after irradiation. Adult group; before irradiation and 1-144 h after irradiation) or Welch's t-test (two-tailed) (Juvenile group; 144 h after irradiation.) was performed according to the results of the F-test via the Microsoft Excel. **P* < 0.05 compared to the SE group.

(C, D) Changes in γ-H2AX-positive cell rate over time up to 3 h after X-ray 2 Gy irradiation in liver tissue of juvenile SE / EE (C) or adult SE / EE (D) mice. The data show mean ± SEM; n = 3 mice per group. The statistical approach is Student's t-test (two-tailed) (Juvenile group; before irradiation and 1, 3 h after irradiation. Adult group; before irradiation and 1, 3 h after irradiation) was performed according to the results of the F-test via the Microsoft Excel. **P* < 0.05 compared to the SE group.

(E, F) Changes in the rate of decrease in γ-H2AX-positive cells after X-ray 2 Gy irradiation in liver tissue of juvenile SE / EE (E) or adult SE / EE (F) mice. It is a relative value to the γ-H2AX-positive cell rate detected 1 h after irradiation. The data show mean ± SEM; n = 3 mice per group. The statistical approach is Student's t-test (two-tailed) (Juvenile group; 1-24 h after irradiation. Adult group; 1-144 h after irradiation) or Welch's t-test (two-tailed) (Juvenile group; 144 h after irradiation.) was performed according to the results of the F-test via the Microsoft Excel. **P* < 0.05 compared to the SE group.

(G, H) Changes in the rate of decrease in γ-H2AX-positive cells up to 6 h after irradiation with X-ray 2 Gy in liver tissue of juvenile SE / EE (G) or adult SE / EE (H) mice. The data show mean ± SEM; n = 3 mice per group. The statistical approach is Student's t-test (two-tailed) (Juvenile group; 1-6 h after irradiation. Adult group; 1-6 h after irradiation) was performed according to the results of the F-test via the Microsoft Excel. **P* < 0.05 compared to the SE group.


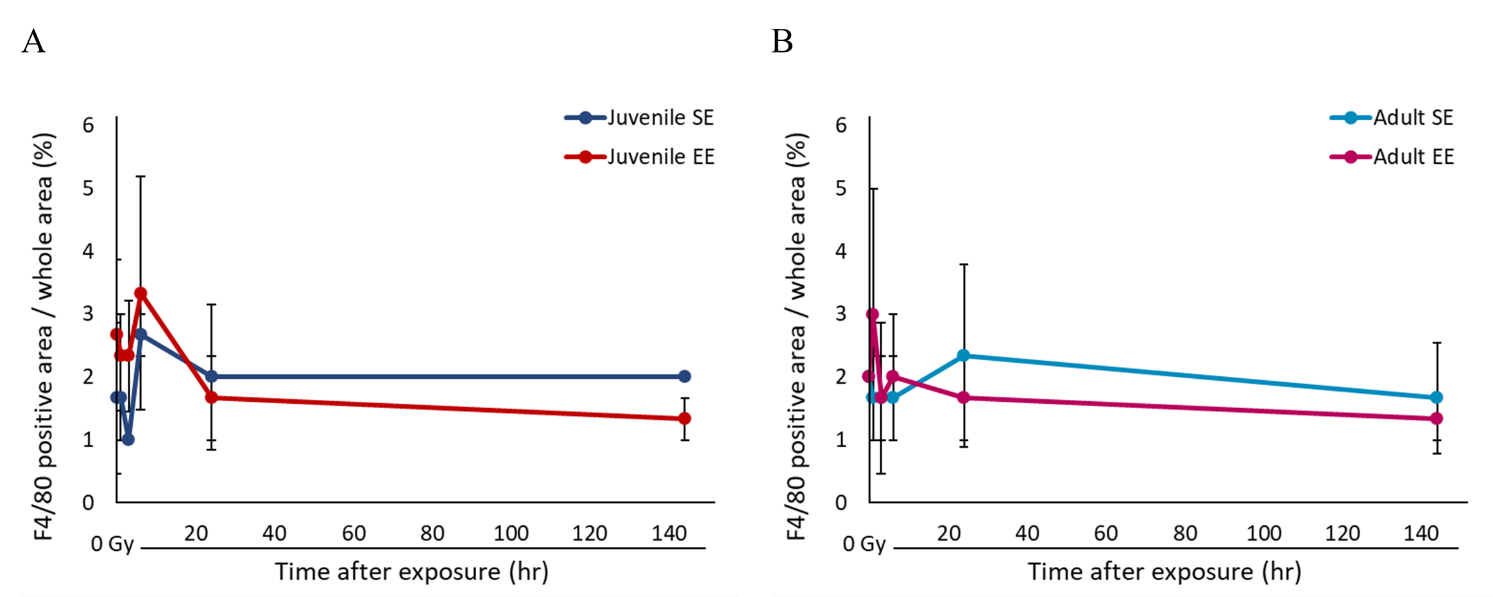


## Supplementary Figure 2. Effect of EE on inflammatory response by activated　macrophages in liver tissue.

(A, B) Changes in F4/80 positive intensity over time after X-ray non-irradiation and 2 Gy irradiation in liver tissue of juvenile SE / EE (A) or adult SE / EE (B) mice. The data show mean ± SEM; n = 3 mice per group. The statistical approach is Student's t-test (two-tailed) (Juvenile group; before irradiation and 1, 6, 24 h after irradiation. Adult group; before irradiation and 1-144 h after irradiation) or Welch's t-test (two-tailed) (Juvenile group; 3, 144 h after irradiation.) was performed according to the results of the F-test via the Microsoft Excel. **P* < 0.05 compared to the SE group.
